# Supplementary material for: Direction of actin flow dictates integrin LFA-1 orientation during leukocyte migration
Source: Nat Commun. 2017 Dec 11;8:2047. doi: 10.1038/s41467-017-01848-y (PMC5725580; doi:10.1038/s41467-017-01848-y)
Supplement: Supplementary file 1 — Supplementary Information [file 41467_2017_1848_MOESM1_ESM.pdf]

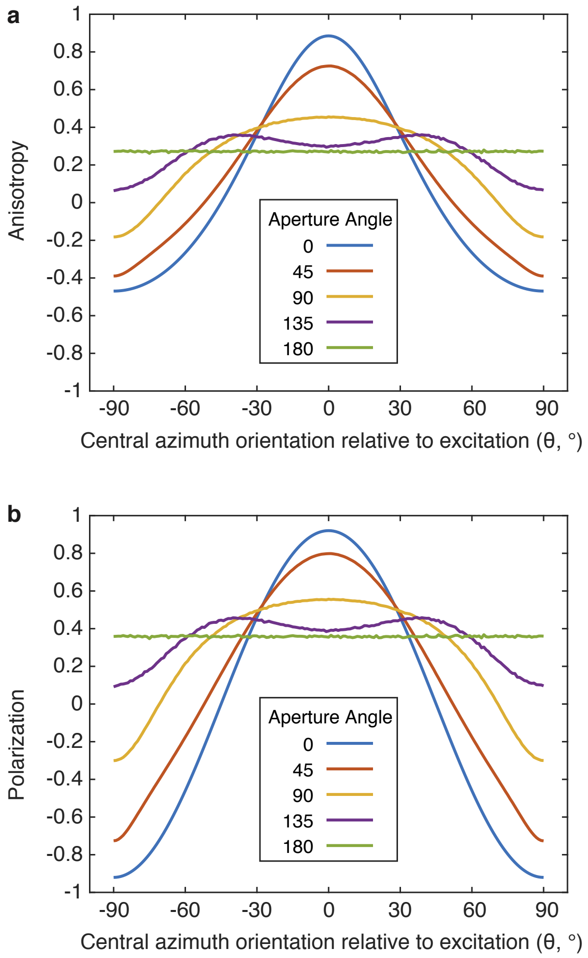

Supplementary Figure 1. Simulation of EA-TIRFM response to constrained dipoles.
a-b. Ensembles of dipoles with differing spreads around an average orientation (aperture angles of 0, 45, 90, 135, and 180°) are excited by light polarized at different angles θ relative to the central azimuth of the dipole ensemble. The emission is collected by a 1.49 NA objective and analyzed parallel and perpendicular to the excitation axis.
a. Simulated anisotropy $r = (I_{\parallel}-I_{\perp})/(I_{\parallel}-2I_{\perp})$.
b. Simulated polarization $p = (I_{\parallel}-I_{\perp})/(I_{\parallel}-I_{\perp})$.

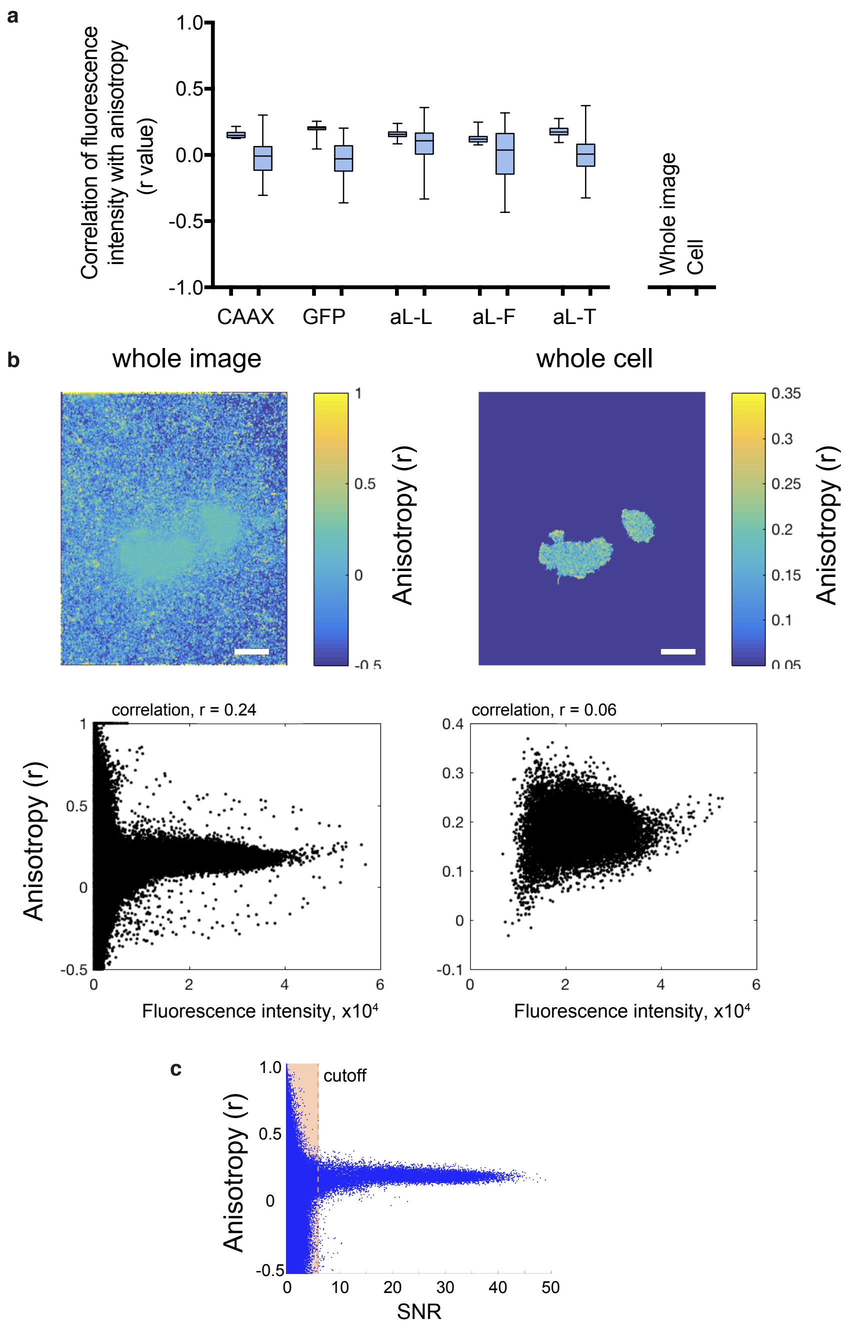

Supplementary Figure 2. Correlation of anisotropy with fluorescence intensity or signal to noise ratio (SNR).
a. Correlation r values between total fluorescence intensity (combined polarization channels, 2x perpendicular + 1x parallel) and anisotropy. Correlation values are shown for whole images (left box) and segmented cells from images (right box). Box plots show the full range (whiskers) of the data and 25-75% range (boxes) with median as line. From left, number of images N = 33, 55, 206, 185, and 36.
b. Representative image showing anisotropy (upper) and plot of total fluorescence intensity versus anisotropy (lower), with whole image data (left) and segmented cell data from the same image (right). Note difference in anisotropy scales. Scale bars are 5 μm.
c. Representative plot of anisotropy vs SNR in a non-segmented image. At low SNR, the anisotropy values are uncertain and therefore an SNR cutoff threshold (SNR>5) was used for all EA-TIRFM analysis.

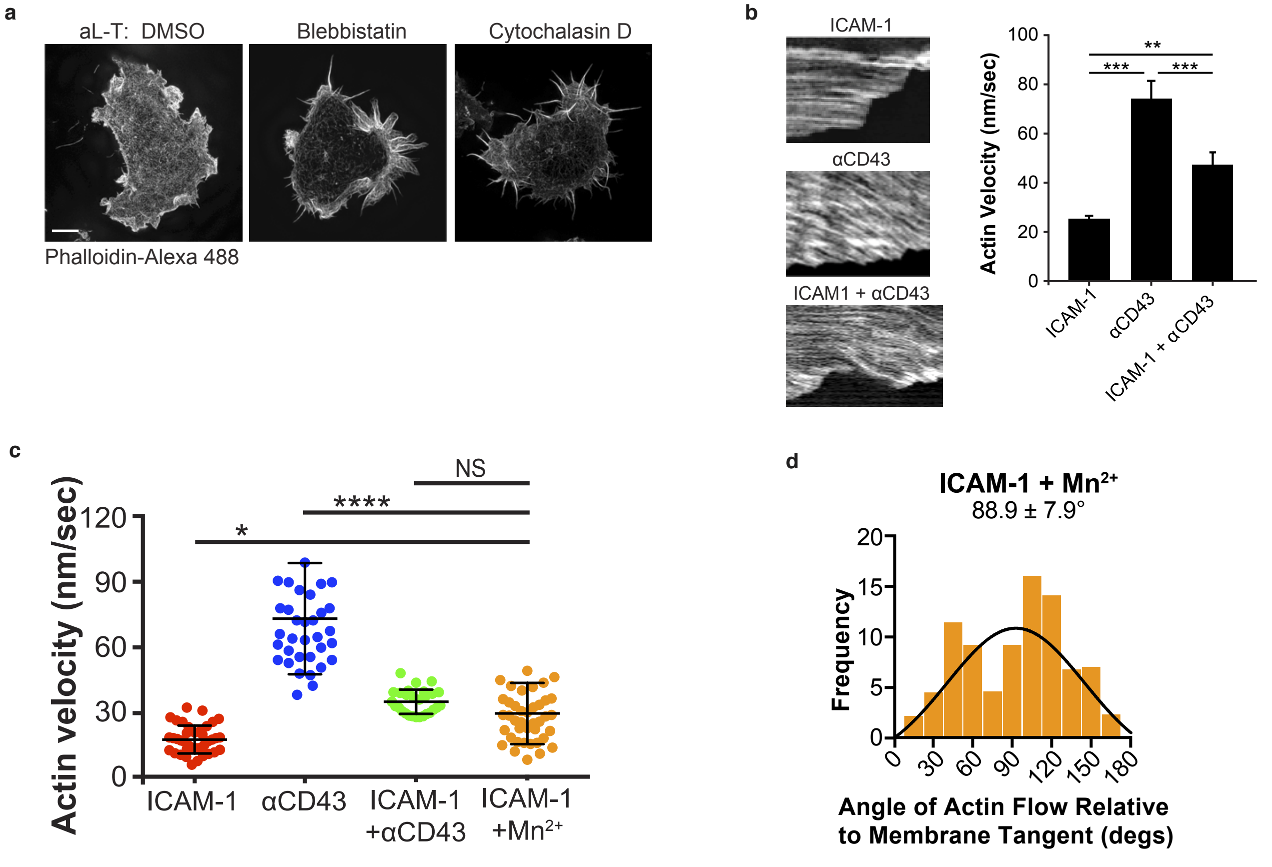

Supplementary Figure 3. Additional actin data.

a. Representative super-resolution SIM images of the actin cytoskeleton in fixed Jurkat T cells migrating on ICAM-1 as labeled with Alexa 488-phalloidin treated with DMSO, 100 μM blebbistatin or 100 nm cytochalasin D. Scale bar = 2 μm.
b. Kymograph analysis of retrograde actin flow and representative kymographs (see Methods)
c. Leading edge actin flow velocity from optical flow analysis. Plots show full range of the data. Bars show mean ± SD. Two-tailed Mann-Whitney tests; $p < 0.5$, $p < 0.0001$ (****). ICAM-1, N=39; αCD43 N=38; and ICAM-1+ anti-CD43, N=25; ICAM-1+Mn^{2+}, N=41. Some data shown in Fig. 3 are repeated here for comparison.
d. Actin flow direction relative to tangent of leading edge membrane. Bins of 15° are shown with gaussian fit and mean ± SEM. N=42.

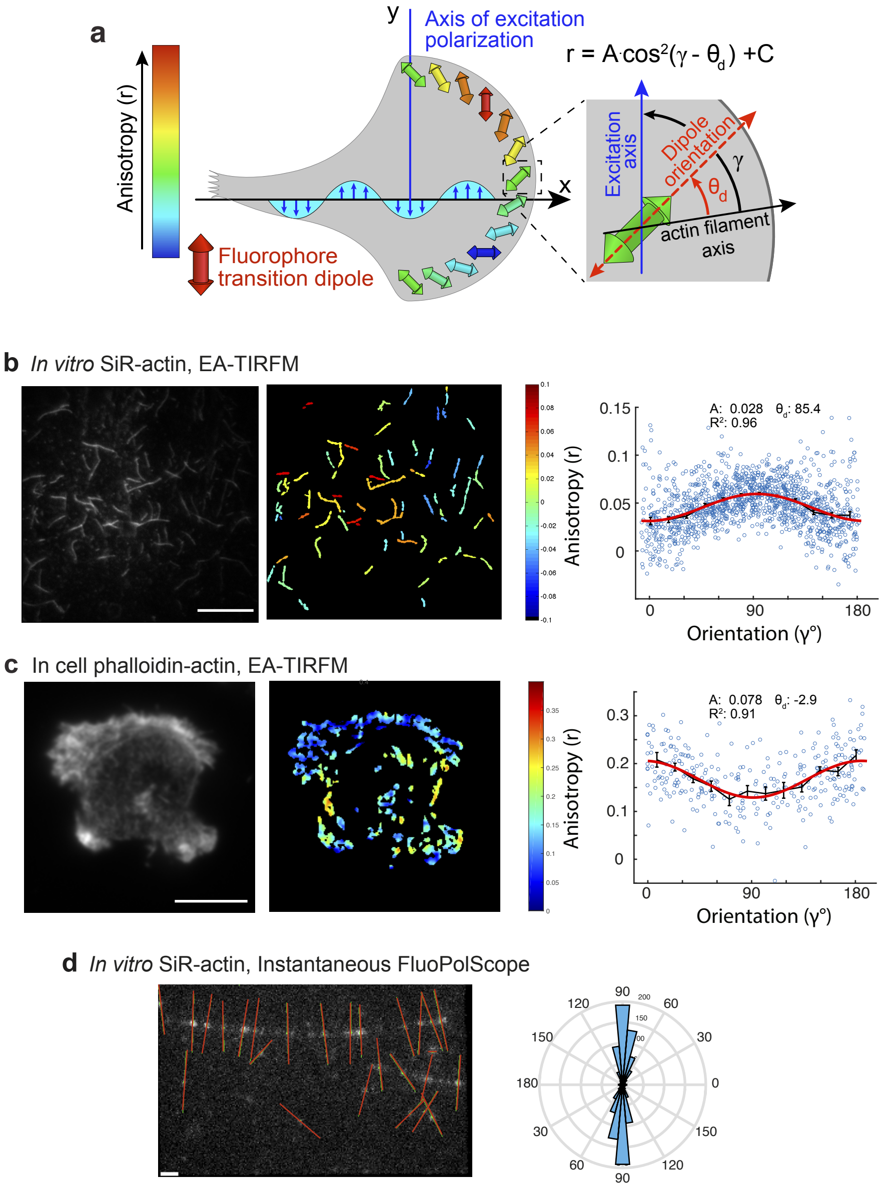

Supplementary Figure 4. Orientation of actin filaments determined with EA-TIRFM and Instantaneous PolScope
a. Schematic showing relation between excitation polarization, transition dipole orientation, and emission anisotropy in EA-TIRFM modified from Fig. 4a. The inset equation was used to fit EA-TIRFM data in b and c where r is anisotropy, A is the amplitude in angular dependence of anisotropy, the angles γ and θd are defined relative to the long axis of actin filaments, and C is a constant.
b. Left, representative example of *in vitro* SiR-actin filaments with fluorescence, anisotropy and scatter plot of anisotropy vs orientation angle with data on filaments from 15 images using EA-TIRFM. Right, running average is shown as a black line and the fit curve using cosine function (see Methods and panel a) is shown in red. Scale bar is 10 μm.
c. Representative example of actin phalloidin in fixed Jurkat T cells with total fluorescence (left), anisotropy (middle) and plot of anisotropy vs orientation angle (right) with data from 6 cells using EA-TIRFM, running average shown as a black line, and fitted curve using cosine function (panel a) shown in red. Scale bar is 5 μm.
d. Left, lines indicate ensemble dipole orientation of SiR-actin probe bound within different segments of actin filaments in vitro using Instantaneous FluoPolScope. Right, angular distribution of the ensemble dipole orientation relative to the filament orientation (θ_d). Radial axis is the number of filaments which fall within each angular bin. Mean orientation ± full width at half-maximum: 91.4° +-14°. N (number of filaments analyzed) = 643. Scale bar is 1 μm.

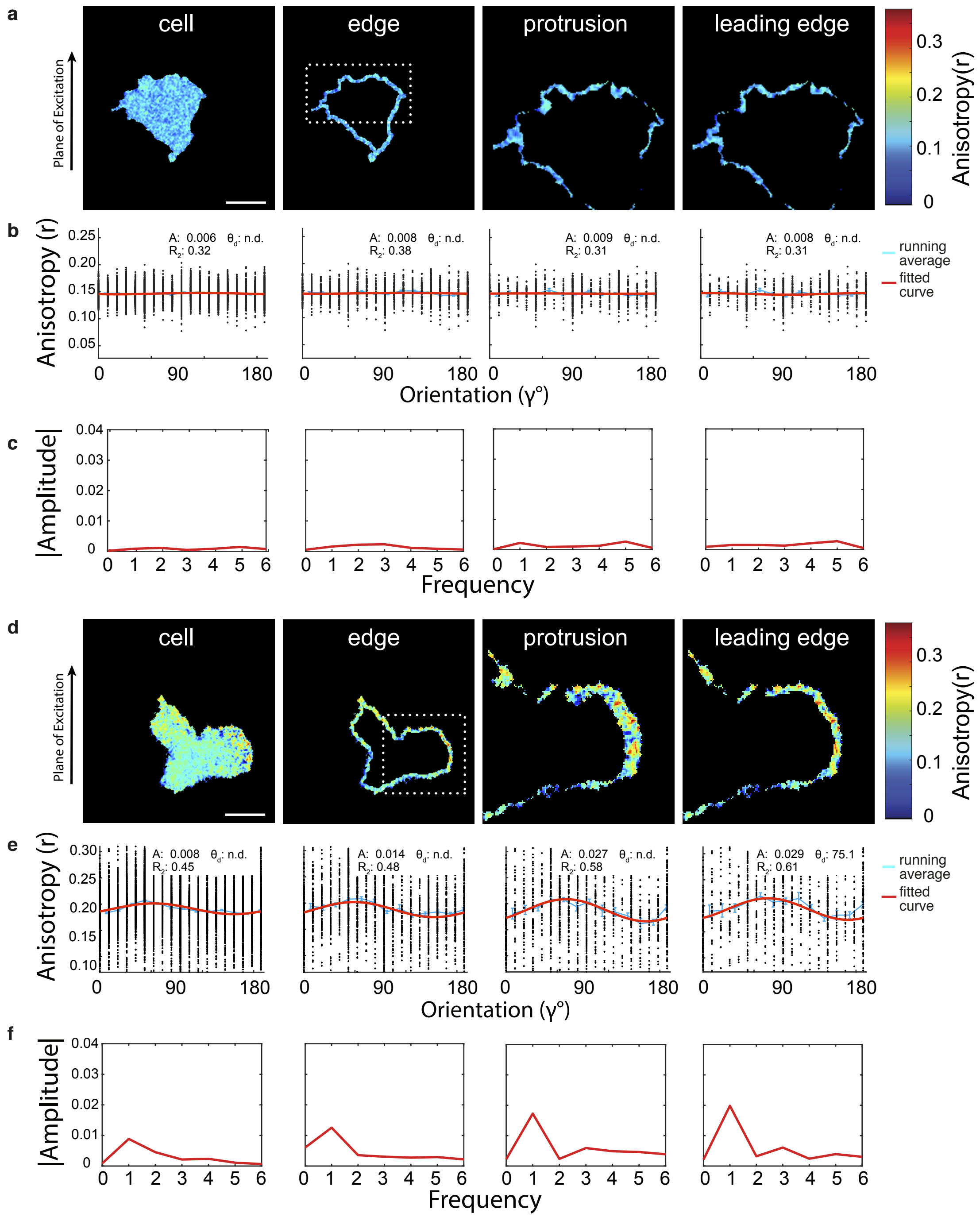

Supplementary Figure 5. Angular dependence analysis – Cytosolic GFP (a-c) and αL-F-GFP (d-f)

a, d. Representative example of migrating Jurkat T cell expressing cytosolic GFP (a) and αL-F-GFP (d), respectively. Each cell is segmented into whole cell, edge, protrusion and leading edge regions (see Methods). Scale bar is 5 μm.
b, e. Scatter plot of anisotropy vs orientation angle with data from a and d. Running average is shown as a blue line and a fitted curve using cosine function (see Methods) is shown in red with mean values from fit above. See Extended Data Table 2 for tabulated values.
c, f. Power spectrum plot from Fourier transform of data from b and e. Absolute amplitude of frequency peaks is plotted on y-axis.

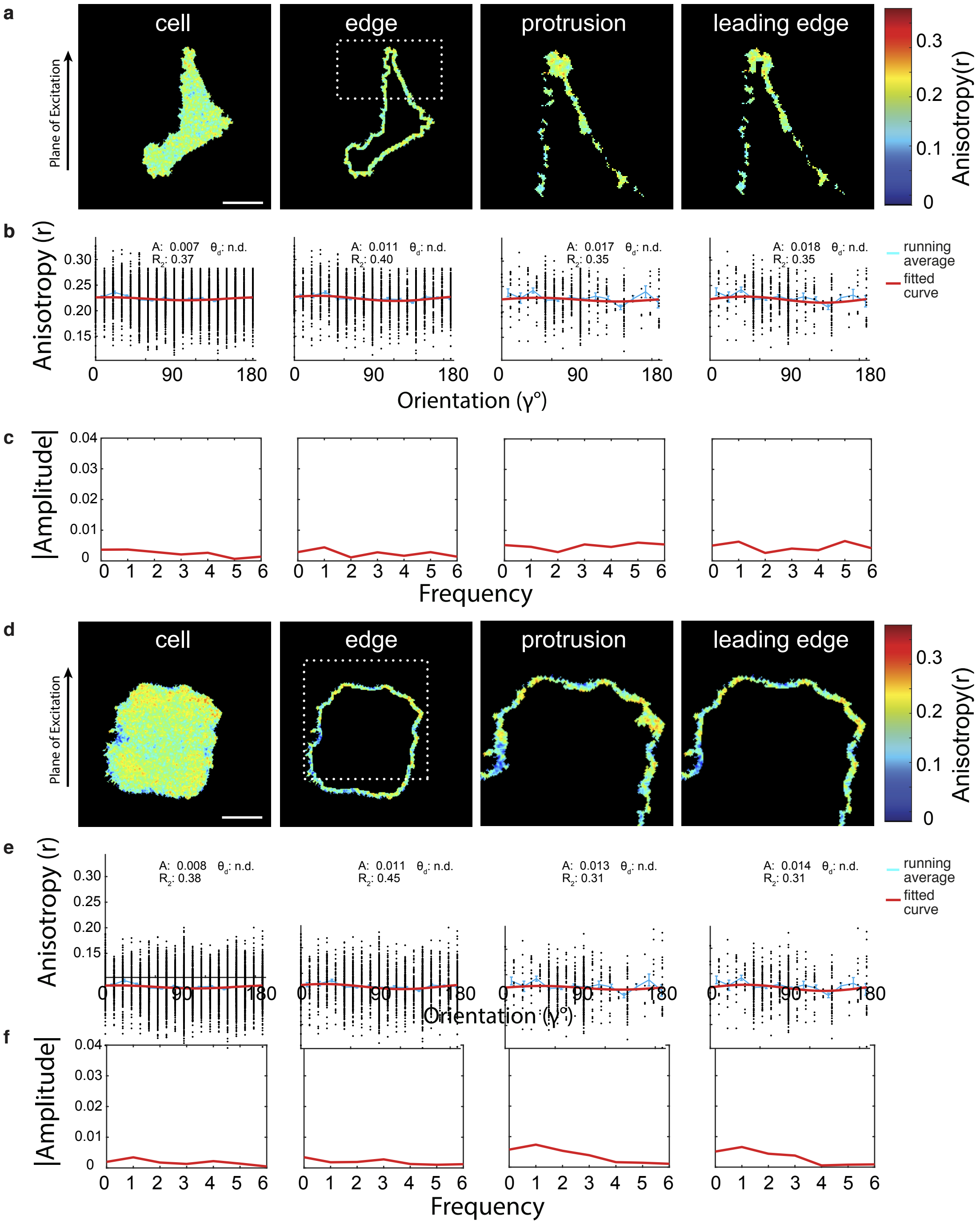

Supplementary Figure 6. Angular dependence analysis – αL-T-GFP with Mn^{2+} (a-c) or on anti-CD43 (d-f)

a, d. Representative example of migrating Jurkat T cell expressing αL-T-GFP with Mn^{2+} (a) or on anti-CD43 (d), respectively. Each cell is segmented into whole cell, edge, protrusion and leading edge regions (see Methods). Scale bar is 5 μm.
b, e. Scatter plot of anisotropy vs orientation angle with data from a and d. Running average is shown as a blue line and a fitted curve using cosine function (see Methods) is shown in red with mean values from fit above. See Extended Data Table 2 for tabulated values.
c, f. Power spectrum plot from Fourier transform of data from b and e. Absolute amplitude of frequency peaks is plotted on y-axis.

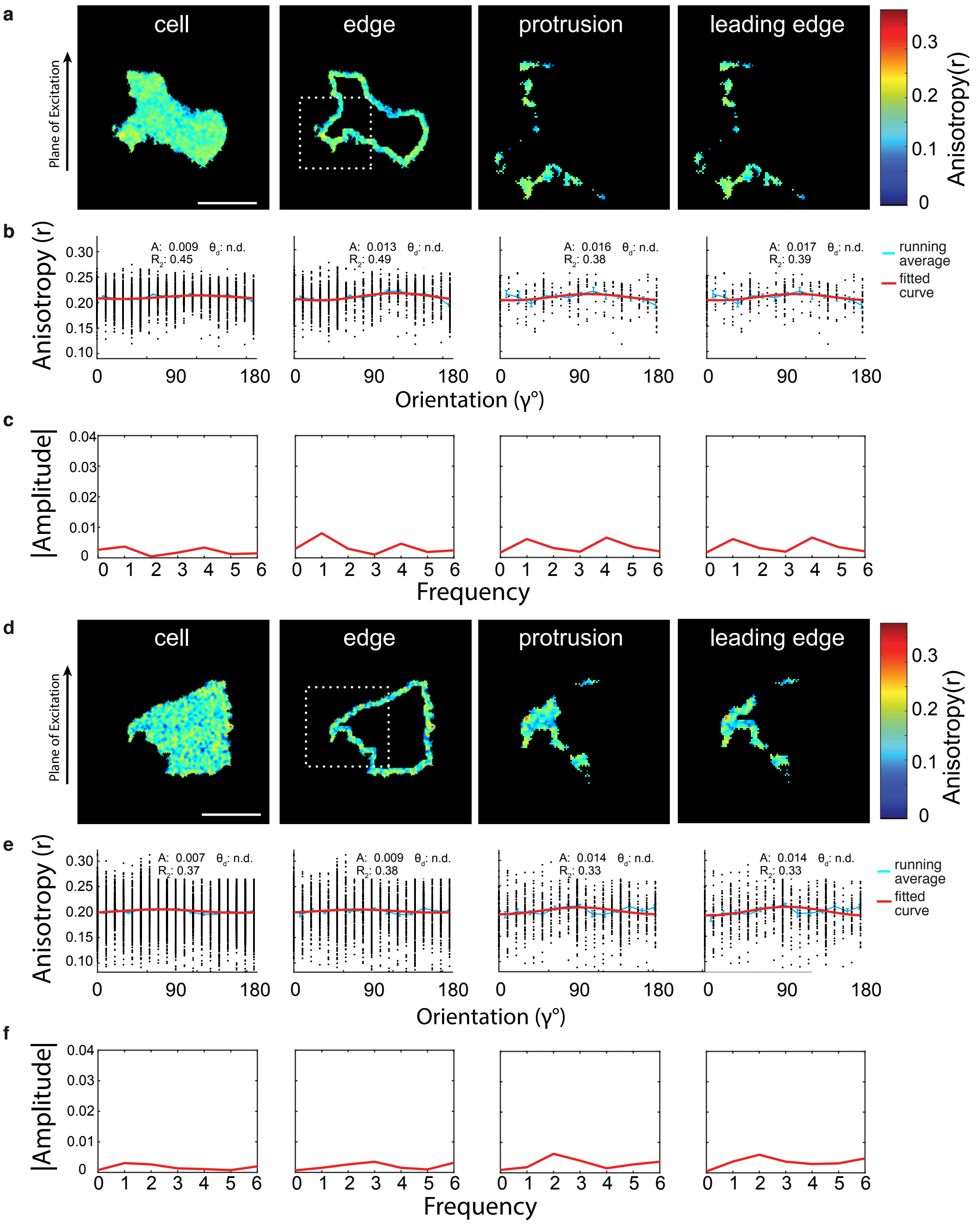

Supplementary Figure 7. Angular dependence analysis – talin head overexpression with αL-F-GFP (a-c) and αL-T-GFP (d-f)

a, d. Representative example of migrating Jurkat T cell overexpressing talin head with αL-F-GFP (a) or αL-T-GFP (d). Each cell is segmented into whole cell, edge, protrusion and leading edge regions (see Methods). Scale bar is 5 μm.
b, e. Scatter plot of anisotropy vs orientation angle with data from a and d. Running average is shown as a blue line and a fitted curve using cosine function (see Methods) is shown in red with mean values from fit above. See Extended Data Table 2 for tabulated values.
c, f. Power spectrum plot from Fourier transform of data from b and e. Absolute amplitude of frequency peaks is plotted on y-axis.

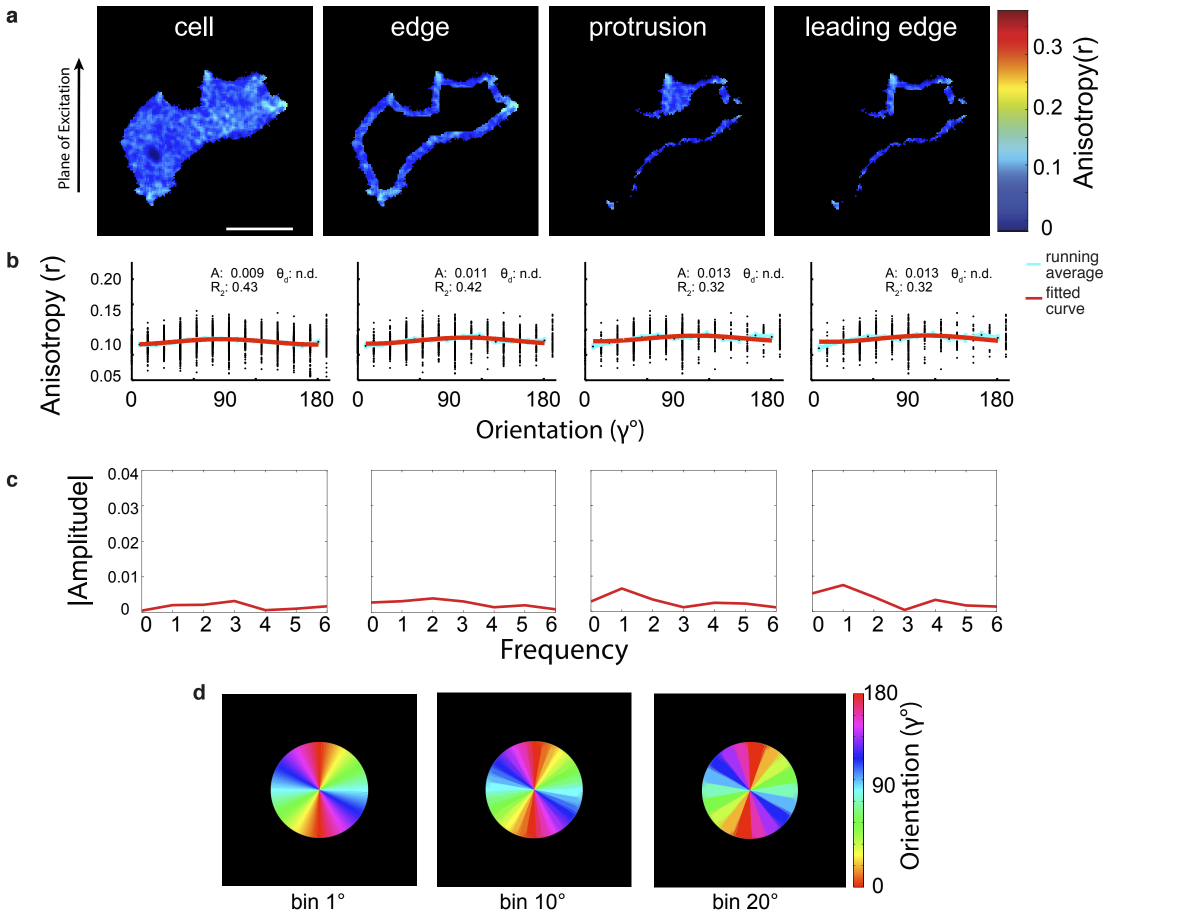

Supplementary Figure 8. Angular dependence analysis of CAAX and orientation algorithm simulation data.

a. Representative example of migrating Jurkat T cell expressing CAAX-GFP. The cell is segmented into whole cell, edge, protrusion and leading edge regions (see Methods). Scale bar is 5 μm.
b. Scatter plot of anisotropy vs orientation angle with data from a and d. Running average is shown as a blue line and a fitted curve using cosine function (see Methods) is shown in red with mean values from fit above. See Extended Data Table 2 for tabulated values.
c. Power spectrum plot from Fourier transform of data from b. Absolute amplitude of frequency peaks is plotted on y-axis.
d.The images show three circles where relative orientation was determined with orientation values binned to 1, 10 or 20 degrees. The algorithm is described in the Methods section.

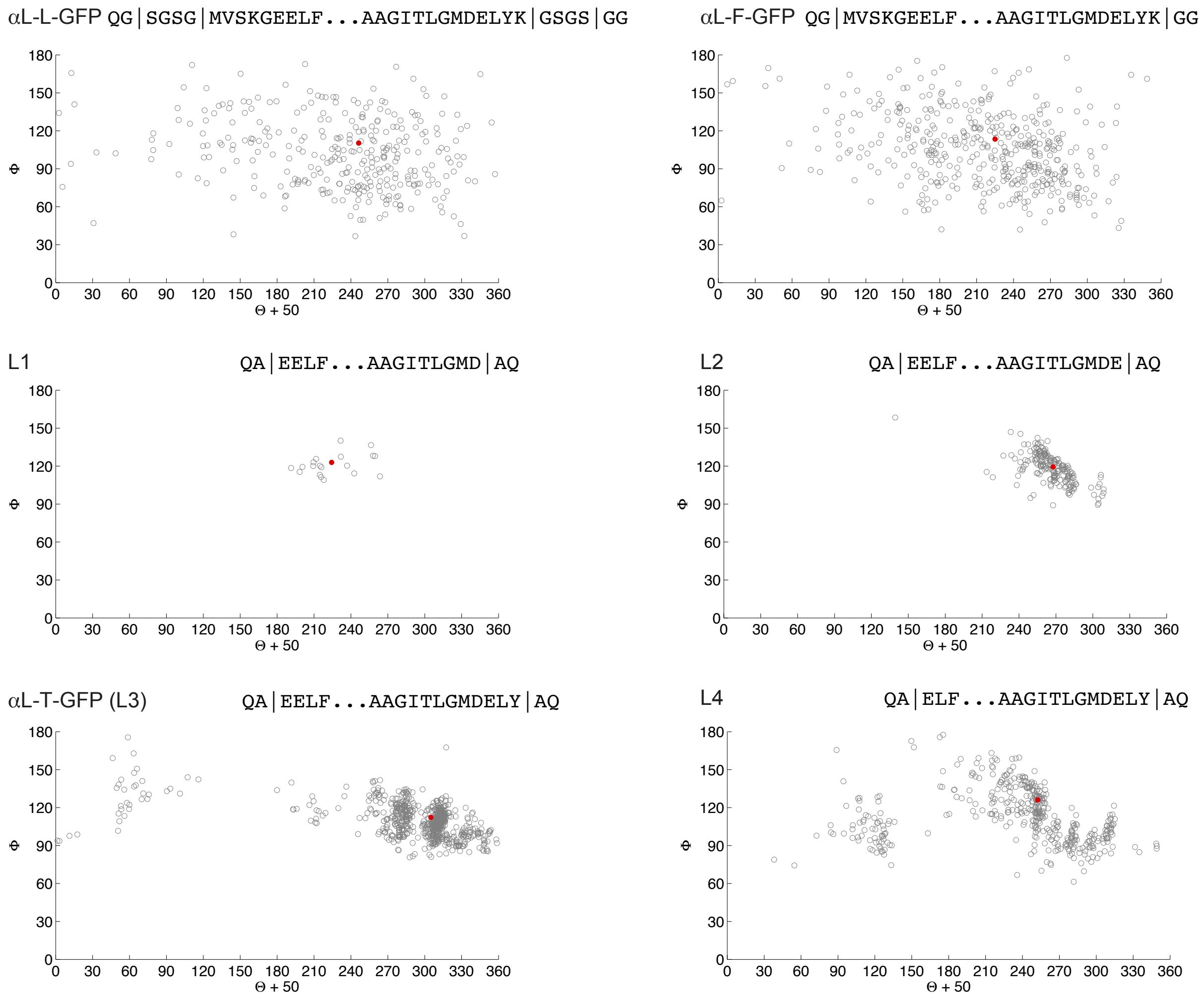

Supplementary Figure 9. Transition dipole orientation in Rosetta ensembles: dependence on residues included at the GFP-integrin fusion junctions.

Open circles show GFP dipole orientations in the integrin-microscope frame of reference for Rosetta ensemble members. The red dot is the centroid of all ensembles. Residues at integrin, linker, and GFP junctions are shown above each plot, with construct names as described in Extended Data Table 1.

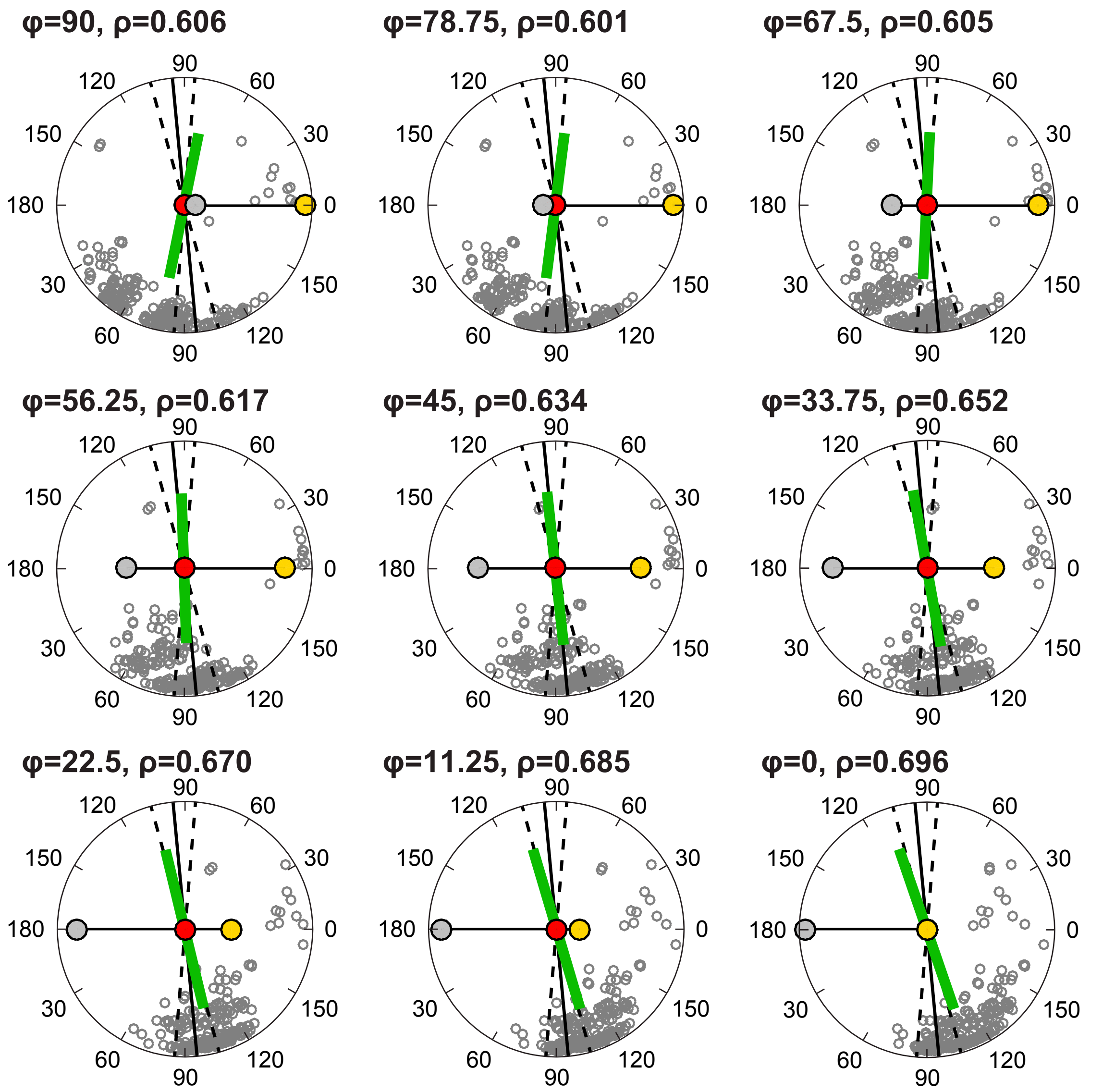

Supplementary Figure 10. Effect of aL-T tilt on dipole orientation in the XY plane of the integrin-microscope reference frame.

GFP transition dipole orientation for each Rosetta ensemble member (lowest 40% in energy) is represented by its intersection with the surface of a sphere in spherical coordinates in the integrin-microscope reference frame. The projection of each ensemble member in the XY plane of the reference frame at θ = 0 and ϕ at the indicated tilt relative to the Z axis is shown as an open circle. The calculated ensemble transition dipole is shown as a green line with orientation θ and length scaled to the polarization factor such that *p*=1 at the radius of the sphere that is projected as a circle. Red, gold, and silver circles represent the projected positions of the three integrin atoms that define the reference frame and correspond to key ligand, α-leg junction, and β-leg junction residues (see main text Fig. 6b). FluoPolScope experimental dipole projection measurements are shown with mean orientation (solid black line) ± 1 sd (dashed black lines). Note: each dipole projection is shown only in one direction from the origin to represent the asymmetry of the GFP molecule in which the dyad-symmetric dipole is present. To represent this asymmetry of GFP, the projections include θ values from 0 to 360°. However, the dyad symmetry of the dipole in the plots is represented by graphing two series of θ values from 0 to 180°, and by reflecting the calculated and experimentally observed transition dipoles.

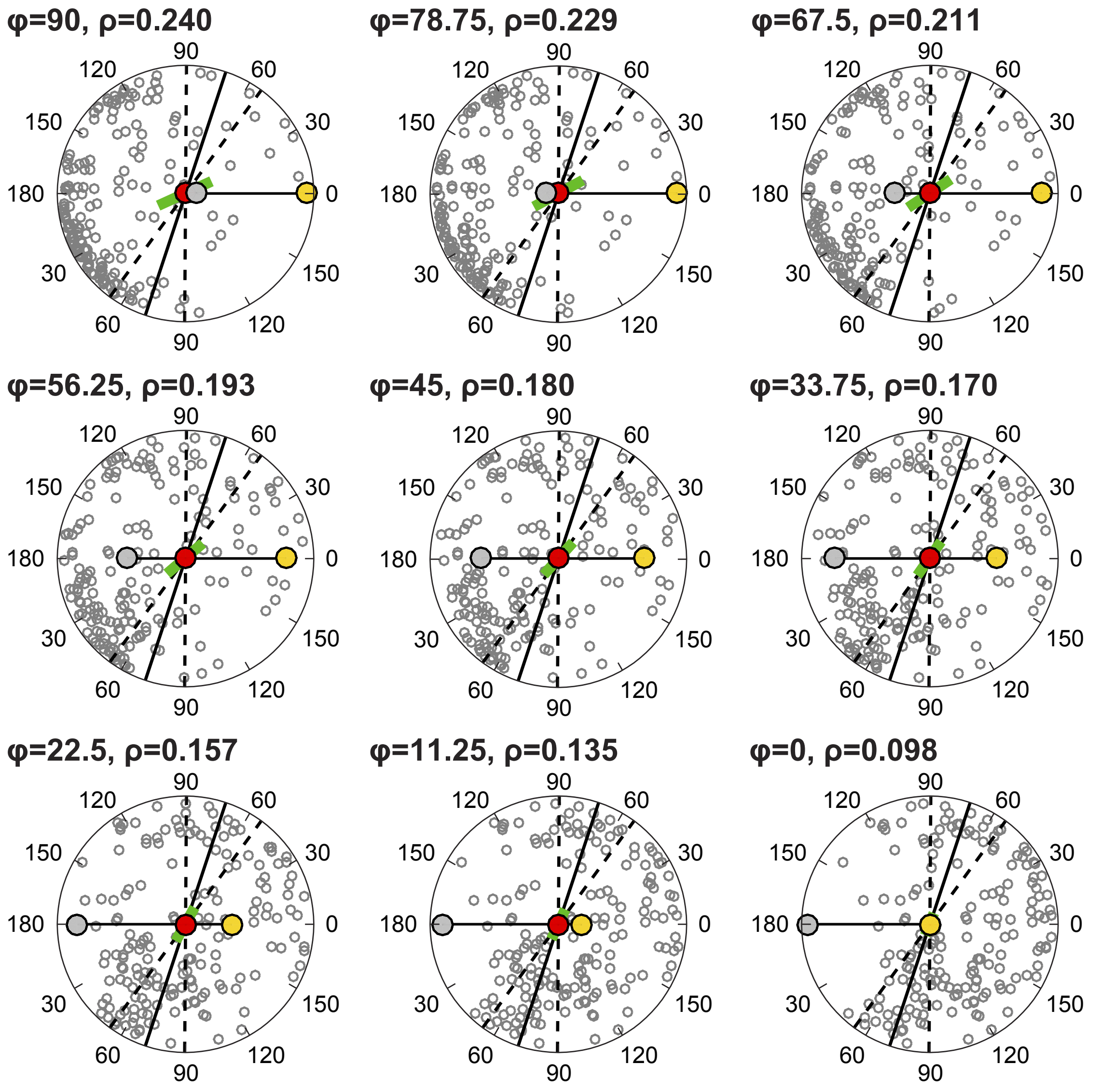

Supplementary Figure 11. Effect of aL-F tilt on dipole orientation in the XY plane of the integrin-microscope reference frame.

GFP transition dipole orientation for each Rosetta ensemble member (lowest 40% in energy) is represented by its position on the surface of a sphere in spherical coordinates in the integrin-microscope reference frame. The projection of each ensemble member in the XY plane of the reference frame at θ = 0 and ϕ at the indicated tilt relative to the Z axis is shown as an open circle. The calculated ensemble transition dipole is shown as a green line with orientation θ and length scaled to the polarization factor such that *p*=1 at the radius of the sphere that is projected as a circle. Red, gold, and silver circles represent the projected positions of the three integrin atoms that define the reference frame and correspond to key ligand, α-leg junction, and β-leg junction residues (see main text Fig. 6b). FluoPolScope experimental dipole projection measurements are shown with mean orientation (solid black line) ± 1 sd (dashed black lines). Note: each dipole projection is shown only in one direction from the origin to represent the asymmetry of the GFP molecule in which the dyad-symmetric dipole is present. To represent this asymmetry of GFP, the projections include θ values from 0 to 360°. However, the dyad symmetry of the dipole in the plots is represented by graphing two series of θ values from 0 to 180°, and by reflecting the calculated and experimentally observed transition dipoles.

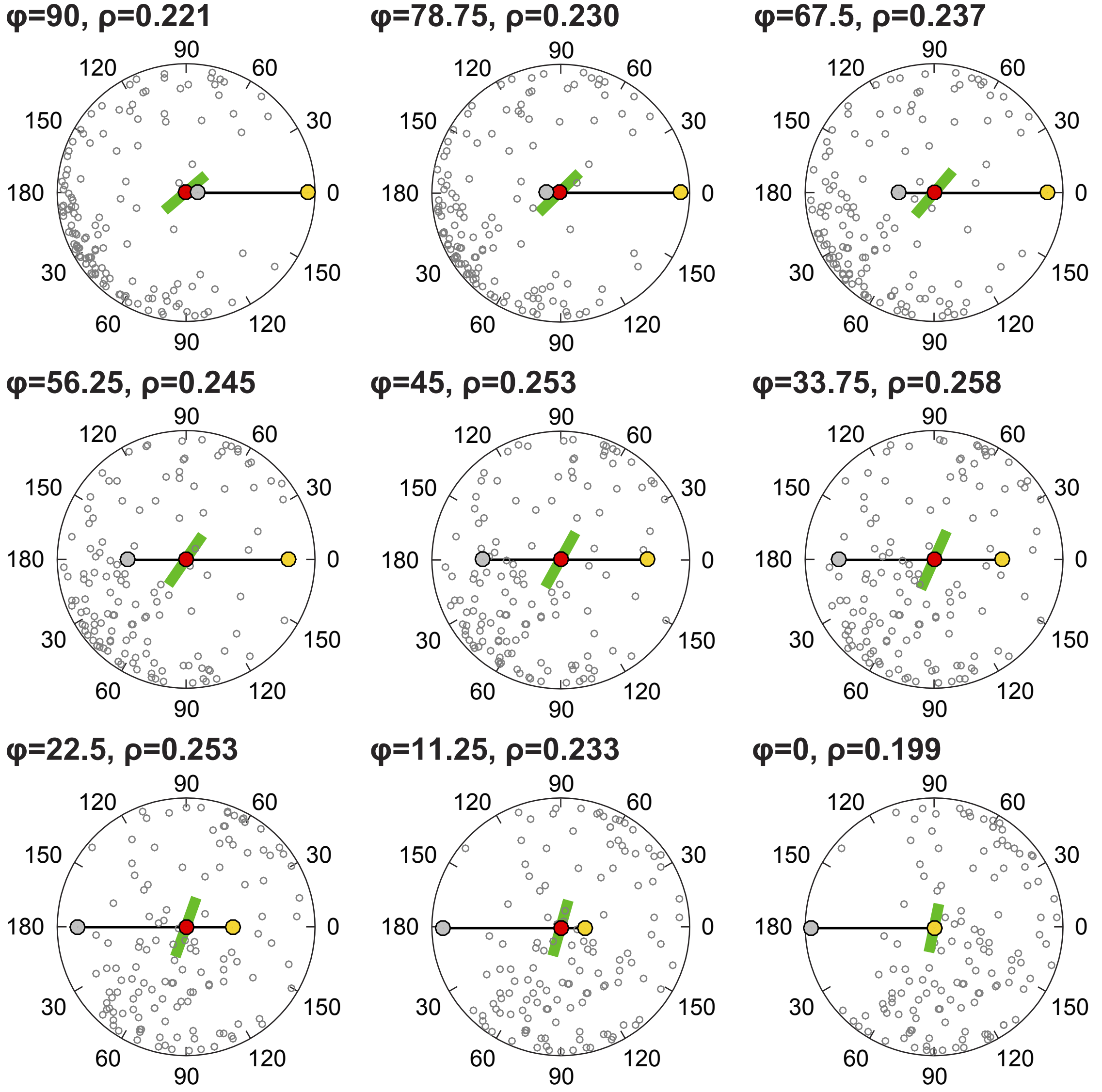

Supplementary Figure 12. Effect of aL-L tilt on dipole orientation in the XY plane of the integrin-microscope reference frame.

GFP transition dipole orientation for each Rosetta ensemble member (lowest 40% in energy) is represented by its position on the surface of a sphere in spherical coordinates in the integrin-microscope reference frame. The projection of each ensemble member in the XY plane of the reference frame at θ = 0 and ϕ at the indicated tilt relative to the Z axis is shown as an open circle. The calculated ensemble transition dipole is shown as a green line with orientation θ and length scaled to the polarization factor such that p=1 at the radius of the sphere that is projected as a circle. Red, gold, and silver circles represent the projected positions of the three integrin atoms that define the reference frame and correspond to key ligand, α-leg junction, and β-leg junction residues (see main text Fig. 6b). FluoPolScope experimental dipole projection measurements are shown with mean orientation (solid black line) ± 1 sd (dashed black lines). Note: each dipole projection is shown only in one direction from the origin to represent the asymmetry of the GFP molecule in which the dyad-symmetric dipole is present. To represent this asymmetry of GFP, the projections include θ values from 0 to 360°. However, the dyad symmetry of the dipole in the plots is represented by graphing two series of θ values from 0 to 180°, and by reflecting the calculated and experimentally observed transition dipoles.

Supplementary Table 1. List of αL-GFP constructs

Published name	Full name	Working name	αL	linker	GFP	linker	αL
	αL-EGFP_C1		...LLFQEPQA		EELF...AAGIT		AQHWSQ...
	αL-EGFP_C2		...LLFQEPQA		EELF...AAGITL		AQHWSQ...
	αL-EGFP_C3		...LLFQEPQA		EELF...AAGITLG		AQHWSQ...
	αL-EGFP_C4		...LLFQEPQA		EELF...AAGITLGM		AQHWSQ...
	αL-EGFP_C5	L1	...LLFQEPQA		EELF...AAGITLGMD		AQHWSQ...
	αL-EGFP_C6	L2	...LLFQEPQA		EELF...AAGITLGMDE		AQHWSQ...
	αL-EGFP_C7		...LLFQEPQA		EELF...AAGITLGMDEL		AQHWSQ...
αL-T-GFP	αL-EGFP_C8	L3	...LLFQEPQA		EELF...AAGITLGMDELY		AQHWSQ...
	αL-EGFP_C9		...LLFQEPQA		EELF...AAGITLGMDELYK		AQHWSQ...
	αL-EGFP_C8_N3	L4	...LLFQEPQA		ELF...AAGITLGMDELY		AQHWSQ...
	αL-EGFP_N1		...LLFQEPQA		KAEELF...AAGITLG		AQHWSQ...
	aL-EGFP_N2		...LLFQEPQA		AEELF...AAGITLG		AQHWSQ...
	aL-EGFP_N3		...LLFQEPQA		ELF...AAGITLG		AQHWSQ...
	aL-EGFP_N4		...LLFQEPQA		LF...AAGITLG		AQHWSQ...
	aL-EGFP_N5		...LLFQEPQA		F...AAGITLG		AQHWSQ...
αL-F-GFP	αL-EGFP_FL	aL	...LLFQEPQG		MVSKGEELF...AAGITLGMDELYK		GGHWSQ...
αL-L-GFP	αL-EGFP_LL	aLL	...LLFQEPQG	SGSG	MVSKGEELF...AAGITLGMDELYK	GSGS	GGHWSQ...

List of all constructs used for either imaging experiments or Rosetta simulations.

Supplementary Table 2 | Summary of Ensemble Dipole Orientations and Polarization Factors

		Tilt (φ)								
**Construct**	**Ensemble value**	**90**	**78.75**	**67.5**	**56.25**	**45**	**33.75**	**22.5**	**11.25**	**0**
αL-GFP-L	Transition dipole orientation (θ)	40.4	44.5	49.8	55.5	61.1	66.1	70.3	74.2	78.2
	Polarization factor (ρ)	0.22	0.23	0.24	0.24	0.25	0.26	0.25	0.23	0.19
αL-GFP-F	Transition dipole orientation (θ)	24.5	28.4	33.5	40.4	47.9	55.1	60.9	65.2	68.5
	Polarization factor (ρ)	0.24	0.23	0.21	0.19	0.18	0.17	0.16	0.13	0.09
αL-GFP-T	Transition dipole orientation (θ)	78.4	82.8	87.3	91.9	96.2	100.1	103.6	106.6	108.9
	Polarization factor (ρ)	0.61	0.60	0.61	0.62	0.63	0.65	0.67	0.69	0.69

Supplementary Table 3. Summary of data obtained by fitting anisotropy (r) vs geometric orientation

Construct	Condition		N cell/ protrusion	Expt.	1 cell SNR	2 edge SNR	3 prot SNR	4 l. edge SNR	1 cell A	2 edge A	3 prot A	4 l. edge A	1 cell R2	2 edge R2	3 protl. R2	4 edge R2	4 θ_d	refined N	4 refined R2	4 refined θ_d
αL-GFP-T	(10 μg/ml) sICAM-1		54 / 206	9														23 / 53		
		median			17.1	12.7	11.9	11.5	0.009	0.014	0.029	0.033	0.44	0.52	0.63	0.65	101.4		0.72	104.5
		mean			19.3	14.0	13.3	12.7	0.011	0.016	0.033	0.036	0.44	0.50	0.60	0.64	97.4		0.70	98.5
		S.D.			8.3	5.9	5.8	5.3	0.008	0.011	0.019	0.019	0.27	0.23	0.19	0.17	40.5		0.13	37.6
αL-GFP-T	(10 μg/ml) sICAM-1 + 1 mM Mn^{2+}		22 / 85	3																
		median			18.2	13.2	11.9	11.8	0.006	0.010	0.015	0.016	0.37	0.39	0.36	0.37	n.d.			
		mean			19.5	14.2	12.9	12.6	0.007	0.011	0.017	0.018	0.37	0.40	0.35	0.35	n.d.			
		S.D.			7.9	5.6	5.2	5.0	0.005	0.008	0.013	0.013	0.20	0.22	0.22	0.21	n.d.			
αL-GFP-T	anti-CD43 (10 μg/ml)		15 / 52	5																
		median			13.8	10.1	9.2	9.1	0.007	0.010	0.012	0.011	0.37	0.48	0.32	0.30	n.d.			
		mean			14.5	10.9	10.2	10.0	0.008	0.011	0.013	0.014	0.38	0.45	0.31	0.31	n.d.			
		S.D.			4.2	3.1	3.5	3.3	0.005	0.006	0.008	0.010	0.24	0.24	0.19	0.21	n.d.			
αL-GFP-T	(10 μg/ml) sICAM-1 + talin head overexpression		15 / 58	3																
		median			18.5	13.1	12.1	11.9	0.007	0.008	0.012	0.012	0.37	0.39	0.30	0.28	n.d.			
		mean			18.3	13.3	13.1	12.5	0.007	0.009	0.014	0.014	0.37	0.38	0.33	0.33	n.d.			
		S.D.			5.5	4.1	4.6	4.3	0.005	0.006	0.009	0.009	0.24	0.24	0.25	0.25	n.d.			
αL-GFP-F	(10 μg/ml) sICAM-1		38 / 185	5														28 / 63		
		median			24.0	16.0	14.2	13.1	0.009	0.012	0.024	0.026	0.45	0.53	0.60	0.63	70.2		0.68	68.9
		mean			29.3	20.7	19.0	18.2	0.008	0.014	0.027	0.029	0.45	0.48	0.58	0.61	75.1		0.64	75.7
		S.D.			9.3	9.1	7.7	7.1	0.006	0.008	0.014	0.014	0.23	0.22	0.19	0.15	45.3		0.15	46.6
αL-GFP-F	(10 μg/ml) sICAM-1 + talin head overexpression		13 / 71	2																
		median			26.4	16.6	13.2	12.9	0.008	0.012	0.013	0.015	0.47	0.52	0.36	0.39	n.d.			
		mean			26.4	17.3	14.8	14.3	0.009	0.013	0.016	0.017	0.45	0.49	0.38	0.39	n.d.			
		S.D.			8.8	6.9	6.1	5.8	0.006	0.008	0.014	0.013	0.24	0.24	0.22	0.23	n.d.			
cytosolic GFP	(20 μg/ml) sICAM-1		15 / 55	3																
		median			123.9	88.4	81.7	79.0	0.004	0.007	0.007	0.008	0.28	0.38	0.31	0.29	n.d.			
		mean			177.7	126.6	121.5	115.9	0.006	0.008	0.009	0.008	0.32	0.38	0.31	0.31	n.d.			
		S.D.			156.9	115.9	112.0	107.4	0.005	0.005	0.005	0.004	0.20	0.23	0.20	0.18	n.d.			
membrane GFP (CAAX)	(20 μg/ml) sICAM-1		14 / 83	5																
		median			27.9	23.3	22.6	21.9	0.008	0.009	0.012	0.012	0.42	0.44	0.29	0.30	n.d.			
		mean			61.9	46.6	44.0	41.9	0.009	0.011	0.013	0.013	0.43	0.42	0.32	0.32	n.d.			
		S.D.			82.2	59.7	54.5	51.2	0.004	0.006	0.007	0.007	0.22	0.25	0.21	0.22	n.d.			

1 = whole cell, 2 = cell edge, 3 = protrusion, 4 = leading edge
A = amplitude
R2 = goodness-of-fit

θ_d = phase shift for leading edge using circular statistics. Only determined for aL-T and aL-F on sICAM under normal condition. Refined indicates a manual selection of leading edges with a clear arc-like shape.
