# Supplementary material for: Direction of actin flow dictates integrin LFA-1 orientation during leukocyte migration
Source: Nat Commun. 2017 Dec 11;8:2047. doi: 10.1038/s41467-017-01848-y (PMC5725580; doi:10.1038/s41467-017-01848-y)
Supplement: Supplementary file 3 — Description of Additional Supplementary Files [file 41467_2017_1848_MOESM3_ESM.docx]

**Description of Additional Supplementary Files**

File Name: Supplementary Movie 1

Description: Structured illumination microscopy movie of the actin cytoskeleton visualized with lifeact-mNeonGreen in Jurkat T cells migrating on ICAM-1 (10 µg/ml). Optical actin flow vector maps of the same cells are shown on the right. Vectors encode flow direction by color (circular keys in the direction from center of circle to perimeter) and velocity by length. See main text Figure 3 for velocity scale. 1 frame per ~1.3 seconds.

File Name: Supplementary Movie 2

Description: Structured illumination microscopy movie of the actin cytoskeleton visualized with lifeact-mNeonGreen in Jurkat T cells migrating on anti-CD43 IgG (10 µg/ml). Optical actin flow vector maps of the same cells are shown on the right. Vectors encode flow direction by color (circular keys in the direction from center of circle to perimeter) and velocity by length. See main text Figure 3 for velocity scale. 1 frame per ~1.3 seconds.

File Name: Supplementary Movie 3

Description: Structured illumination microscopy movie of the actin cytoskeleton visualized with lifeact-mNeonGreen in Jurkat T cells migrating on a mixture of ICAM-1 and anti-CD43 IgG (10 µg/ml each). Optical actin flow vector maps of the same cells are shown on the right. Vectors encode flow direction by color (circular keys in the direction from center of circle to perimeter) and velocity by length. See main text Figure 3 for velocity scale. 1 frame per ~1.3 seconds.

File Name: Supplementary Movie 4

Description: EA-TIRFM movie of a migrating Jurkat T cell on ICAM-1 (10 µg/ml) showing emission anisotropy of expressed GFP-LFA-1 fusion αL-T. Time is shown in upper left corner. 1 frame per 10 seconds.

File Name: Supplementary Movie 5

Description: EA-TIRFM movie of a migrating Jurkat T cell on ICAM-1 (10 µg/ml) showing emission anisotropy of cytoplasmic expressed GFP. Time is shown in upper left corner. 1 frame per 15 seconds.
